# Impact of Pathological Stratification on the Clinical Outcomes of Advanced Well-Differentiated/Dedifferentiated Liposarcoma Treated with Trabectedin

**DOI:** 10.3390/cancers13061453

**Published:** 2021-03-22

**Authors:** Chiara Fabbroni, Giovanni Fucà, Francesca Ligorio, Elena Fumagalli, Marta Barisella, Paola Collini, Carlo Morosi, Alessandro Gronchi, Angelo Paolo Dei Tos, Paolo Giovanni Casali, Roberta Sanfilippo

**Affiliations:** 1 Adult Mesenchymal Tumour Medical Oncology Unit, Fondazione IRCCS Istituto Nazionale dei Tumori, 20133 Milan, Italy; chiara.fabbroni@istitutotumori.mi.it (C.F.); giovanni.fuca@istitutotumori.mi.it (G.F.); francesca.ligorio@istitutotumori.mi.it (F.L.); Elena.fumagalli@istitutotumori.mi.it (E.F.); paolo.casali@istitutotumori.mi.it (P.G.C.); 2 Department of Pathology, Fondazione IRCCS Istituto Nazionale dei Tumori, 20133 Milan, Italy; marta.barisella@istitutotumori.mi.it (M.B.); paola.collini@istitutotumori.mi.it (P.C.); 3 Department of Radiology, Fondazione IRCCS Istituto Nazionale dei Tumori, 20133 Milan, Italy; carlo.morosi@istitutotumori.mi.it; 4 Department of Surgery, Fondazione IRCCS Istituto Nazionale dei Tumori, 20133 Milan, Italy; alessandro.gronchi@istitutotumori.mi.it; 5 Department of Pathology and Molecular Genetics, Treviso General Hospital, 31100 Treviso, Italy; angelo.deitos@unipd.it; 6 Department of Medicine, University of Padova School of Medicine, 35128 Padova, Italy; 7 Oncology and Hemato-Oncology Department, University of Milan, 20122 Milan, Italy

**Keywords:** trabectedin, liposarcoma, grading, soft tissue sarcoma, chemotherapy

## Abstract

**Simple Summary:**

The treatment options for advanced well differentiated liposarcoma (WDLPS) and dedifferentiated liposarcoma (DDLPS) are scant, especially after the failure of first-line, anthracycline-based treatment, when trabectedin is one of the most active agents currently approved. Thus, the identification of biological characteristics allowing an efficient patients stratification in this setting appears mandatory. Here, applying a grading-based stratification, we showed that trabectedin may be more active against low-grade LPS (i.e., WDLPS and low-grade DDLPS) than in high-grade LPS (i.e., high-grade DDLPS). If confirmed, our data might allow the implementation of grading as a tool patients’ stratification in this setting.

**Abstract:**

Background. We previously showed that grading can prognosticate the outcome of retroperitoneal liposarcoma (LPS). In the present study, we aimed to explore the impact of pathological stratification using grading on the clinical outcomes of patients with advanced well-differentiated LPS (WDLPS) and dedifferentiated LPS (DDLPS) treated with trabectedin. Patients: We included patients with advanced WDLPS and DDLPS treated with trabectedin at the Fondazione IRCCS Istituto Nazionale dei Tumori between April 2003 and November 2019. Tumors were categorized in WDLPS, low-grade DDLPS, and high-grade DDLPS according to the 2020 WHO classification. Patients were divided in two cohorts: Low-grade (WDLPS/low-grade DDLPS) and high-grade (high-grade DDLPS). Results: A total of 49 patients were included: 17 (35%) in the low-grade cohort and 32 (65%) in the high-grade cohort. Response rate was 47% in the low-grade cohort versus 9.4% in the high-grade cohort (logistic regression *p* = 0.006). Median progression-free survival (PFS) was 13.7 months in the low-grade cohort and 3.2 months in the high-grade cohort. Grading was confirmed as an independent predictor of PFS in the Cox proportional-hazards regression multivariable model (adjusted hazard ratio low-grade vs. high-grade: 0.45, 95% confidence interval: 0.22–0.94; adjusted *p* = 0.035). Conclusions: In this retrospective case series, sensitivity to trabectedin was higher in WDLPS/low-grade DDLPS than in high-grade DDLPS. If confirmed in larger series, grading could represent an effective tool to personalize the treatment with trabectedin in patients with advanced LPS.

## 1. Introduction

Liposarcoma (LPS) represents the most common soft tissue sarcoma (STS) of adults, accounting for up to 25% of all cases [1,2]. Based on specific and distinct histological, molecular and clinical characteristics, LPS can be classified into different subtypes that include well-differentiated LPS (WDLPS), dedifferentiated LPS (DDLPS), myxoid/round cell LPS, and pleomorphic LPS. WDLPS and DDLPS represent the two most common subtypes [3].

WDLPS is known to be a low-grade, locally aggressive neoplasm occurring most frequently in the limbs, and with a lower frequency in the retroperitoneum, mediastinum, and head and neck region [4,5,6,7,8]. A risk of distant metastasization does exist and depends on the development of dedifferentiation across relapses. The prognosis of patients with WDLPS is influenced by tumor location. A higher disease-specific mortality was observed for retroperitoneal WDLPS since the tendency to a higher recurrence rate and subsequent risk of dedifferentiation and metastasization [4,5]. DDLPS usually originates in the retroperitoneum, arising de novo in 90% of cases. In the remaining 10% of cases, DDLPS develops as a dedifferentiated recurrence of a previous WDLPS [3]. Notably, the prognostic significance of the extension of the dedifferentiated component in DDLPS is not clear [9]. The metastatic potential of DDLPS is reported to be around 10–50%, with a disease-specific mortality reported to be six-fold higher than in WDLPS [5,10,11]. Treatment options for patients with unresectable and/or metastatic WDLPS and DDLPS are limited. Usually, clinical trials enrolling patients with advanced WDLPS/DDLPS do not provide data about subgroup analysis in the two specific subtypes; thus, there are almost no differences in the clinical management of advanced WDLPS/DDLPS that are considered as a single clinical entity. As for other STS subtypes, standard first-line therapy consists in anthracycline-based chemotherapy [12,13,14]. However, the response rate reported in WDLPS and DDLPS for anthracycline-based treatment is lower, thus leading to traditionally consider this subgroup of STS as a poorly chemo-sensitive subtype [15,16].

After the failure of first-line treatment, trabectedin is one of the most active agents currently approved [17]. Unfortunately, no biomarkers exist to guide the use of trabectedin in anthracycline-refractory WDLPS/DDLPS, and the overall response rate in such a setting does not exceed 10%, even if a durable disease control is possible [17]. In particular, the subgroup analysis of the clinical trial of trabectedin in leiomyosarcoma and liposarcoma, which are the most sensitive histotype, shows a progression-free survival (PFS) of 3 months in dedifferentiated liposarcoma. A further analysis of the impact of histological grade in the population of dedifferentiated liposarcoma is not available [18]. Thus, the identification of biological characteristics allowing an efficient patient stratification in this setting appears mandatory.

Given such unmet clinical needs, and since we previously reported that grading may allow a prognostic stratification of patients with WDLPS/DDLPS [19], we investigated the impact of pathological stratification on the clinical outcomes of patients with advanced WDLPS/DDLPS treated with trabectedin.

## 2. Materials and Methods

### 2.1. Study Population

We retrospectively included all the consecutive patients with advanced WDLPS and DDLPS who started their treatment with trabectedin at the Fondazione IRCCS Istituto Nazionale Tumori, Milan, Italy, from April 2003 to April 2020. Histological diagnosis was centrally reviewed by an expert pathologist. Tumors were categorized in WDLPS, low-grade DDLPS, and high-grade DDLPS according to the 2020 WHO classification [3]. Patients were divided in two cohorts: Low-grade (WDLPS or low-grade DDLPS) and high-grade (high-grade DDLPS). We also reported on the clinical outcomes of a patient with a double DDLPS component: A low-grade DDLPS laterocervical localization and a high-grade DDLPS abdominal localization. Trabectedin was started at the dose of 1.5–1.3 mg/m^2^ in a 24-h continuous infusion, with a steroid premedication the day before treatment and for two days after drug administration. To assess the specificity grading impact on trabectedin outcomes, we evaluated the association grade with the clinical outcomes of previous anthracycline-based chemotherapy in a subgroup of 27 patients with available data as an internal control. All patients signed a written informed consent to the treatment and data collection for research purposes. Patient medical records were reviewed to retrieve demographics, clinical, and pathologic data. The Response Evaluation Criteria in Solid Tumors (RECIST) version 1.1 was used to assess tumor response to treatment [20]. Any radiological reduction in the sum of the longest diameters of target lesions that did not reach the criteria for an objective partial response (PR) was defined as a minor response (MR). Overall response was defined as the presence of PR or MR as best response, whereas disease control was defined as the presence of PR, and MR or a stabilization of disease (SD) as the best response.

### 2.2. Statistical Analysis

Progression-free survival (PFS) was calculated from trabectedin initiation to radiological disease progression or death for any cause. Overall survival (OS) was calculated from trabectedin initiation to death for any cause. Chi-square test, Fisher’s exact or Wilcoxon tests were used, as appropriate, to assess the association between variables. Logistic regression and the Wald test were used to evaluate if grade and other variables were predictors of trabectedin activity. A penalized likelihood method originally proposed by Firth was used to deal with separation in logistic regression [21]. The odds ratio (OR) together with a 95% confidence interval (CI) were provided for logistic regression analyses. The Kaplan–Meier method and Cox proportional-hazards regression model were used for survival analyses. The hazard ratio (HR) together with 95% CI were provided for Cox proportional-hazards regression analyses. Statistical significance threshold was set to a two-tailed 0.05 value. Statistical analyses were performed using R software (version 3.5.0).

## 3. Results

### 3.1. Patients’ Characteristics

A total of 49 patients with WDLPS/DDLPS who started their treatment with trabectedin at our Institution between April 2003 and November 2019 were included: 17 (35%) had a WDLPS (10 patients) or low-grade DDLPS (seven patients) and were included in the low-grade cohort, whereas 32 (65%) had a high-grade DDLPS and were included in the high-grade cohort. Patients’ disease and treatment characteristics are summarized in Table 1.

The median age was 63 years (range: 37–77 years) and tumor location was the retroperitoneum in 46 out of 49 patients (93.9%). Twenty-two out of 49 patients (44.9%) had a locally advanced disease at time of trabectedin initiation, while 27 out of 49 (55.1%) had a metastatic disease. Most patients were ECOG PS 0 (34 out of 49, 69.4%) and received more than two previous lines of systemic therapy (31 out of 49, 63.3%). Patients in the high-grade cohort were more frequently female (*p* = 0.03), with PS ECOG 1–2 (*p* = 0.04) and with a metastatic disease at the time of trabectedin initiation (*p* = 0.001). The median number of cycles received per patient were 4 (range: 1–32) in the entire population, 8 (range: 1–32) for patients in the low-grade cohort, and 4 (range: 1–25) for patients in the high-grade cohort (*p* = 0.008).

### 3.2. Clinical Outcomes According to FNCLCC Grade

Among patients in the low-grade cohort, we observed 6 PR, 2 MR, 8 SD, and 1 progression of disease (PD) as best response, for an overall response rate of 47%. Among patients in the high-grade cohort, we observed 3 PR, 13 SD, and 16 PD, for an overall response rate of 9.4% (Table 2). At a logistic regression analysis, low-grade was a positive predictor of response to trabectedin both at univariate analysis (OR: 8.59, 95% CI: 1.87–39.41; *p* = 0.006) and in the multivariable model including other characteristics associated with response (OR: 5.84, 95% CI: 1.06–32.17; *p* = 0.009) (Table 3).

**Figure 1:**
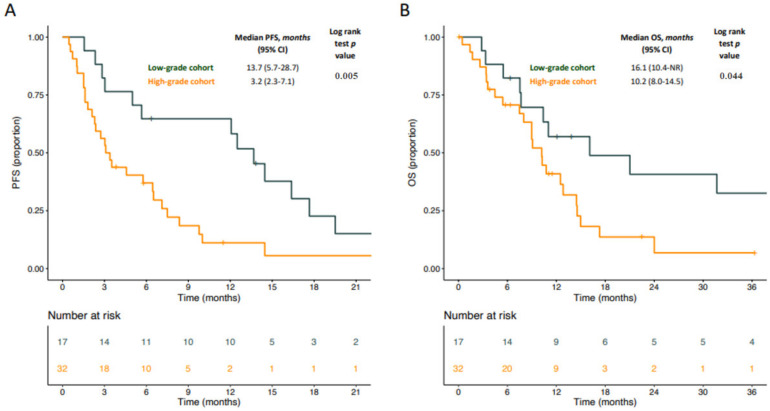
Kaplan–Meier curves for progression-free survival (PFS) (**A**) and OS (**B**) according to pathological stratification. Dark green lines indicate patients in the low-grade cohort whereas orange lines indicate patients in the high-grade cohort.

With a median follow-up time of 36.3 months, median PFS was 13.7 months for patients in the low-grade cohort and 3.2 months for patients in the high-grade cohort (log-rank test *p* = 0.005) (Figure 1A). Of note, we observed a similar PFS for patients with low-grade DDLPS and WDLPS (Figure S1A). A subgroup analysis within the low-grade cohort showed that patients obtaining an overall response with trabectedin had a statistically significant longer PFS (median: 17 months vs. 6 months; log-rank test *p* = 0.015) (Figure S2A).

In the multivariable model including characteristics associated with PFS in the univariate analysis (i.e., ECOG PS), grading was confirmed as an independent predictor of a better PFS (HR low-grade vs. high-grade 0.45; 95% CI, 0.22-0.94; *p* = 0.035) (Table 4).

Patients in the low-grade cohort experienced a longer median OS (16.1 months, 95% CI: 10.4-not reached) compared to patients in the high-grade cohort (10.2 months, 95% CI: 8.0–14.5; log-rank test *p* = 0.044) (Figure 1B). However, in the multivariable model including characteristics associated with OS in the univariate analysis (i.e., age), grading was not confirmed as an independent predictor of OS (HR: 0.54, 95% CI: 0.25–1.18; *p* = 0.13) (Table S1). In line with what observed for PFS, patients with low-grade DDLPS experienced a similar OS compared to patients with WDLPS (Figure S1B). A subgroup analysis within the low-grade cohort showed no difference in terms of OS for patients obtaining an overall response with trabectedin compared to patients not achieving a response (Figure S2B).

We also evaluated the association of grading with the clinical outcomes of previous anthracycline-based chemotherapy in a subgroup of 27 patients with available data, as an internal control. No difference in terms of overall response rate was observed (*p* = 0.287), even if no tumor responses were reported in the low-grade cohort, whereas the overall response rate in the high-grade cohort was 17.6%.

**Figure 2:**
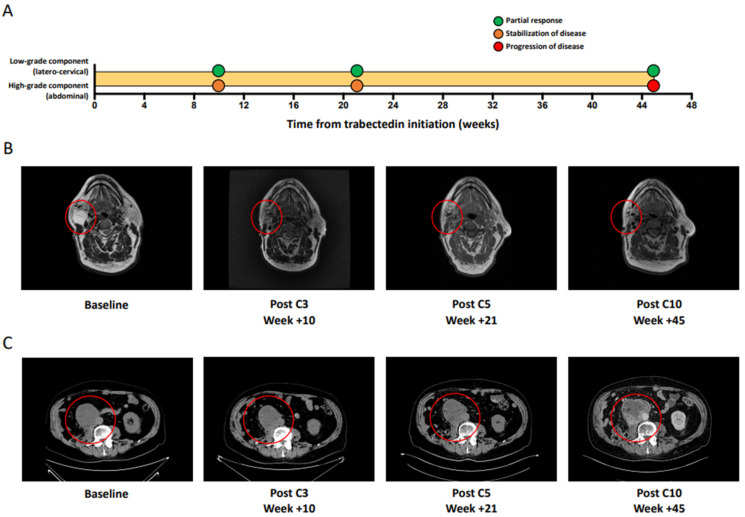
Anecdotal mixed response to trabectedin in a patient with double dedifferentiated liposarcoma (DDLPS) component. (**A**) shows the different dynamic of response to trabectedin of the low-grade, laterocervical component (low-grade DDLPS) vs. the high-grade, abdominal component (high-grade DDLPS). (**B**) (T2 weighted magnetic resonance imaging) and (**C**) (computerized tomography scan) show the RECIST tumor response at specific time-points of the low-grade, laterocervical component and of the high-grade, abdominal component, respectively.

Finally, we anecdotally reported on the case of a patient with a double DDLPS component (Figure S3A) experiencing a mixed response to trabectedin: The laterocervical localization (low-grade DDLPS) showed a PR at the first tumor evaluation that was maintained, whereas the abdominal localization (high-grade DDLPS) showed an SD as the best response and eventually progressed after 45 weeks from trabectedin initiation (Figure 2). Because of the abdominal PD, trabectedin was discontinued. Of note, in the previous line of treatment with doxorubicin, the low-grade laterocervical localization showed a primary resistance (PD as best response after the third cycle) (Figure S3B), whereas the high-grade abdominal remained stable (Figure S3C).

## 4. Discussion

In this series of 49 patients with advanced WDLPS/DDLPS treated with trabectedin and stratified according to grading [3], we observed an overall response rate of 20%, with a median PFS of 13.7 months for the low-grade cohort and of 3.2 months for the high-grade cohort. Furthermore, a partial response was reached in 35% of patients in the low-grade cohort, as compared to 9% of patients in the high-grade cohort. Consistently, since the higher clinical benefit experienced, patients within the low-grade cohort received a higher number of cycles of trabectedin (8 cycles as compared to 4). In contrast, grading was not associated with the clinical outcomes of previous anthracycline-based chemotherapy, as assessed in a subgroup of 27 patients as an internal control, even if all the tumor responses observed were in patients with high-grade DDLPS. Intriguingly, we also observed a differential anti-tumor activity of trabectedin in low-grade DDLPS vs. high-grade DDLPS at an intra-patient level, in a case of DDLPS with a double component. In our series, patients with a WDLP/G1 had a better PFS and OS than patients with a high grade DDLP. This is obviously understandable, since by definition the natural history of the two entities is expected to be, respectively, slower and faster. However, we did not find a better PFS in favor of WDLP in the retrospective analysis of these patients when previously treated with anthracyclines. This would suggest that our results could not be merely explained by a more indolent course of WDLP. Indeed, PFS of the responding patients in the subgroup of WDLP/G1 was higher than for non-responding ones. While clearly this is not evidence per se of the efficacy of trabectedin, it is compatible with a possible effect.

We acknowledge that our study had some limitations, including the limited number of patients included over a 17-year period, and the fact that the grading assessment was made on primary tumors from surgical specimens or diagnostic biopsies. Regarding the latter issue, we are aware that a WDLPS or a low-grade DDLPS may evolve into a high-grade DDLPS and that, though rarely, the opposite may also happen (since a high-grade component may have been successfully controlled with treatments and a subsequent progression may affect only the residual low-grade component). However, even taking into account these caveats, the difference in response rate and PFS observed in the two cohorts was remarkable.

Interestingly, in a retrospective analysis of 28 patients with advanced, pre-treated WDLPS/DDLPS receiving high-dose, continuous-infusion ifosfamide (ciHD-IFX), we previously observed an overall response rate in line with the one reported in the present analysis, but all the tumor responses were observed in patients with high-grade DDLPS (similarly to what observed with anthracycline-based chemotherapy in the present study) [19]. With all the limits of an indirect comparison, our data suggest that there may be a differential activity of trabectedin and ciHD-IFX on the two components of WDLPS/DDLPS. Specifically, trabectedin may be more active in WDLPS and low-grade DDLPS (low-grade LPS), whereas ciHD-IFX may be more active in high-grade DDLPS (high-grade LPS). Indeed, it is reasonable that a highly proliferating neoplasm (i.e., high-grade DDLPS) may respond better to conventional cytotoxic chemotherapy with ifosfamide compared to low-grade tumors. We can also speculate on the reasons why trabectedin might be more active in a low-grade LPS (i.e., WDLPS and low-grade DDLPS). Trabectedin works as an alkylating agent but may also exert a wide range of antitumor biological effects targeting both cancer and tumor microenvironment cells [22]. For example, trabectedin showed an extremely high activity in myxoid LPS, a quite rare subgroup of LPS where the drug seems to have a peculiar mechanism of action [23]. In particular, the pathogenesis of myxoid LPS is associated with the t(12;16)(q13;p11) chromosomal translocation resulting in the expression of the FUS-CHOP product, and trabectedin was shown to be able to displace the FUS-CHOP oncogenic chimera from its target DNA sequences, inducing a process of cell differentiation [22]. Even if the molecular events leading to the development of WDLPS/DDLPS are less clear compared to myxoid LPS, our observations about the pronounced activity of trabectedin in low-grade LPS may represent a proof of concept to prompt future preclinical investigations.

## 5. Conclusions

In conclusion, in this retrospective case series analysis of patients with advanced WDLPS/DDLPS, we observed that trabectedin may be more active against low-grade LPS (i.e., WDLPS and low-grade DDLPS) than in high-grade LPS (i.e., high-grade DDLPS).

The molecular bases behind our clinical observation are largely unknown, and translational research efforts are ongoing.

If confirmed on other series and/or prospective studies, although clinical investigation in so highly selected subgroups is challenging, our data might allow the implementation of grading as an effective tool for patients’ stratification, in a setting where, on average, chemo-sensitivity is quite limited, and chemotherapy is the only effective medical option.

In this scenario, a randomized study of anthracycline versus trabectedin in first line in metastatic or locally advanced WDLPS and low-grade DDLPS would be useful to confirm and further explore our observations.

## Figures and Tables

**Table 1:** Patients’ disease and treatment characteristics.

Characteristics	Low-Grade Cohort (*n* = 17) *n* (%)	High-Grade Cohort (*n* = 32) *n* (%)	Total (*n* = 49) *n* (%)	*p*-Value *
**Age, years** Median (range) ≤65 >65	65 (37–74) 8 (47.1) 9 (52.9)	60 (39–77) 20 (62.5) 12 (37.5)	63 (37–77) 28 (57.1) 21 (42.9)	0.763 0.461
**Sex** Male Female	13 (76.5) 4 (23.5)	14 (43.8) 18 (56.2)	27 (55.1) 22 (44.9)	0.030
**ECOG PS** 0 1–2	15 (88.2) 2 (11.8)	19 (59.4) 13 (40.6)	34 (69.4) 15 (30.6)	0.039
**Disease extension** Locally advanced Metastatic	13 (76.5) 4 (23.5)	9 (28.1) 23 (71.9)	22 (44.9) 27 (55.1)	0.001
**Primary site** Retroperitoneal Other	16 (94.1) 1 (5.9)	30 (93.8) 2 (6.2)	46 (93.9) 3 (6.1)	0.960
**Previous lines of treatment, *n*** ≤2 >2	4 (23.5) 13 (76.5)	14 (43.8) 18 (56.2)	18 (36.7) 31 (63.3)	0.167
**Cycles of trabectedin received, *n*** Median (range)	8 (1–32)	4 (1–25)	4 (1–32)	0.008

* Chi-square test, Fisher’s exact test, or Wilcoxon test, as appropriate. Abbreviations. ECOG PS: Eastern Cooperative Oncology Group performance status.

**Table 2:** Best response according to grading.

Characteristic	Low-Grade Cohort (*n* = 17) *n* (%)	High-Grade Cohort (*n* = 32) *n* (%)	Total (*n* = 49) *n* (%)
**Best response** PR MR SD PD	6 (35.3) 2 (11.8) 8 (47) 1 (5.9)	3 (9.4) 0 (0) 13 (40.6) 16 (50)	9 (18.4) 2 (4.1) 21 (42.8) 17 (34.7)
**Overall response (PR+MR)**	8 (47)	3 (9.4)	11 (22.4)
**Disease Control (PR+MR+SD)**	16 (94.1)	16 (50)	32 (65.3)

Abbreviations. PR: Partial response; MR: Minor response; SD: Stable disease; PD: Progressive disease.

**Table 3:** Logistic regression analyses for objective response.

Characteristics	Univariate Analysis		Multivariable Model	
	OR (95% CI)	*p*-Value	OR (95% CI)	*p*-Value
**Age, years** ≤65 >65	Ref 5.13 (1.16–22.68)	0.031	Ref 7.64 (1.23–47.70)	0.030
**Sex** Male Female	Ref 0.63 (0.16–2.53)	0.520	-	-
**ECOG PS** 0 1–2	Ref 0.43 (0.08-2.28)	0.319	-	-
**Disease extension** Locally advanced Metastatic	Ref 0.22 (0.05–0.96)	0.044	Ref 0.24 (0.04–1.55)	0.135
**Primary site** Retroperitoneal Other	Ref 0.44 (0.003–1.62)	0.593 *	-	-
**Previous lines of treatment, *n*** ≤2 >2	Ref 3.27 (0.62–17.25)	0.162	-	-
**Grading** High-grade cohort Low-grade cohort	Ref 8.59 (1.87–39.41)	0.006	Ref 5.84 (1.06–32.17)	0.043

* Firth’s bias reduction method. Abbreviations. OR: Odds ratio; CI: Confidence interval; Ref: Reference; ECOG PS: Eastern Cooperative Oncology Group performance status.

**Table 4:** Cox proportional-hazards regression models for PFS.

Characteristics	Univariate Analysis		Multivariable Model	
	HR (95% CI)	*p*-Value	HR (95% CI)	*p*-Value
**Age, *years*** ≤65 >65	Ref 0.67 (0.36–1.26)	0.214	-	-
**Sex** Male Female	Ref 1.67 (0.89–3.13)	0.113	-	-
**ECOG PS** 0 1–2	Ref 2.48 (1.28–4.80)	0.007	Ref 1.90 (0.95–3.80)	0.070
**Disease extension** Locally advanced Metastatic	Ref 1.46 (0.77–2.79)	0.250	-	-
**Primary site** Retroperitoneal Other	Ref 1.31 (0.40–4.33)	0.657	-	-
**Previous lines of treatment, *n*** ≤2 >2	Ref 0.75 (0.38–1.46)	0.395	-	-
**Grading** High-grade cohort Low-grade cohort	Ref 0.38 (0.19–0.76)	0.006	Ref 0.45 (0.22–0.94)	0.035

Abbreviations. HR: Hazard ratio; CI: Confidence interval; Ref: Reference; ECOG PS: Eastern Cooperative Oncology Group performance status.

## Data Availability

Not applicable.

## Supplementary Materials

The following are available online at https://www.mdpi.com/2072-6694/13/6/1453/s1, Figure S1. Kaplan–Meier curves for PFS (**A**) and OS (**B**) for WDLPS, low-grade DDLPS, and high-grade DDLPS, Figure S2. Kaplan–Meier curves for PFS (**A**) and OS (**B**) in the low-grade cohort according to overall response to trabectedin, Figure S3. Histological characterization and response to doxorubicin in a patient with double DDLPS component, Table S1. Cox proportional-hazards regression models for OS.

## References

[1] Manji G.A., Schwartz G.K. (2016). Managing Liposarcomas: Cutting Through the Fat. J. Oncol. Pract..

[2] Goldblum J., Weiss S., Folpe A.L. (2013). Enzinger and Weiss’s Soft Tissue Tumors.

[3] Sbaraglia M., Bellan E., Tos A.P.D. (2020). The 2020 WHO Classification of Soft Tissue Tumours: News and perspectives. Pathology.

[4] Weiss S.W., Rao V.K. (1992). Well-Differentiated Liposarcoma (Atypical Lipoma) of Deep Soft Tissue of the Extremities, Retroperitoneum, and Miscellaneous Sites. Am. J. Surg. Pathol..

[5] Coindre J.-M., Pédeutour F., Aurias A. (2009). Well-differentiated and dedifferentiated liposarcomas. Virchows Archiv für Pathologische Anatomie und Physiologie und für Klinische Medizin.

[6] Patil S., Senozan S., Ji B., Chamberlain R.S. (2018). Well-Differentiated Extremity and Retroperitoneal Liposarcoma: A Population based Outcomes Study. Surg. Oncol. Clin. Pract. J..

[7] Mavrogenis A.F., Alberghini M., Letson G.D., Lesensky J., Romagnoli C., Ruggieri P. (2011). Atypical Lipomatous Tumors/Well-differentiated Liposarcomas: Clinical Outcome of 67 Patients. Orthopedics.

[8] Lucas D.R., Nascimento A.G., Sanjay B.K.S., Rock M.G. (1994). Well-differentiated Liposarcoma:The Mayo Clinic Experience with 58 Cases. Am. J. Clin. Pathol..

[9] Mussi C., Collini P., Miceli R., Barisella M., Mariani L., Fiore M., Casali P.G., Gronchi A. (2008). The prognostic impact of dedifferentiation in retroperitoneal liposarcoma. Cancer.

[10] Henricks W.H., Chu Y.C., Goldblum J.R., Weiss S.W. (1997). Dedifferentiated Liposarcoma. Am. J. Surg. Pathol..

[11] Nascimento A.G. (2001). Dedifferentiated liposarcoma. Semin. Diagn. Pathol..

[12] Lee A.T.J., Pollack S.M., Huang P., Jones R.L. (2017). Phase III Soft Tissue Sarcoma Trials: Success or Failure?. Curr. Treat. Opt. Oncol..

[13] Santoro A., Tursz T., Mouridsen H., Verweij J., Steward W., Somers R., Buesa J., Casali P., Spooner D., Rankin E. (1995). Doxorubicin versus CYVADIC versus doxorubicin plus ifosfamide in first-line treatment of advanced soft tissue sarcomas: A randomized study of the European Organization for Research and Treatment of Cancer Soft Tissue and Bone Sarcoma Group. J. Clin. Oncol..

[14] Casali P., Abecassis N., Bauer S., Biagini R., Bielack S., Bonvalot S., Boukovinas I., Bovee J.V.M.G., Brodowicz T., Broto J. (2018). Soft tissue and visceral sarcomas: ESMO–EURACAN Clinical Practice Guidelines for diagnosis, treatment and follow-up. Ann. Oncol..

[15] Jones R.L., Fisher C., Al-Muderis O., Judson I.R. (2005). Differential sensitivity of liposarcoma subtypes to chemotherapy. Eur. J. Cancer.

[16] Italiano A., Toulmonde M., Cioffi A., Penel N., Isambert N., Bompas E., Duffaud F., Patrikidou A., Lortal B., Le Cesne A. (2012). Advanced well-differentiated/dedifferentiated liposarcomas: Role of chemotherapy and survival. Ann. Oncol..

[17] Casali P.G., Sanfilippo R., D’Incalci M. (2010). Trabectedin therapy for sarcomas. Curr. Opin. Oncol..

[18] Patel S., Von Mehren M., Reed D.R., Kaiser P., Charlson J., Ryan C.W., Rushing D., Livingston M., Singh A., Seth R. (2019). Overall survival and histology-specific subgroup analyses from a phase 3, randomized controlled study of trabectedin or dacarbazine in patients with advanced liposarcoma or leiomyosarcoma. Cancer.

[19] Sanfilippo R., Bertulli R., Marrari A., Fumagalli E.R., Pilotti S., Morosi C., Messina A., Tos A.P.D., Gronchi A., Casali P.G. (2014). High-dose continuous-infusion ifosfamide in advanced well-differentiated/dedifferentiated liposarcoma. Clin. Sarcoma Res..

[20] Eisenhauer E.A., Therasse P., Bogaerts J., Schwartz L.H., Sargent D., Ford R., Dancey J., Arbuck S., Gwyther S., Mooney M. (2009). New response evaluation criteria in solid tumours: Revised RECIST guideline (version 1.1). Eur. J. Cancer.

[21] Heinze G., Schemper M. (2002). A solution to the problem of separation in logistic regression. Stat. Med..

[22] Larsen A.K., Galmarini C.M., D’Incalci M. (2016). Unique features of trabectedin mechanism of action. Cancer Chemother. Pharmacol..

[23] Grosso F., Jones R.L., Demetri G.D., Judson I.R., Blay J.-Y., Le Cesne A., Sanfilippo R., Casieri P., Collini P., Dileo P. (2007). Efficacy of trabectedin (ecteinascidin-743) in advanced pretreated myxoid liposarcomas: A retrospective study. Lancet Oncol..

