# Supplementary material for: Impact of Pathological Stratification on the Clinical Outcomes of Advanced Well-Differentiated/Dedifferentiated Liposarcoma Treated with Trabectedin
Source: Cancers (Basel). 2021 Mar 22;13(6):1453. doi: 10.3390/cancers13061453 (PMC8005098; doi:10.3390/cancers13061453)
Supplement: Supplementary file 1 [file cancers-13-01453-s001.pdf]

cancers

MDPI

Supplementary Materials: Impact of Pathological Stratification on the Clinical Outcomes of Advanced Well-Differentiated/De-differentiated Liposarcoma Treated with Trabectedin

Chiara Fabbroni, Giovanni Fucà, Francesca Ligorio, Elena Fumagalli, Marta Barisella, Paola Collini, Carlo Morosi, Alessandro Gronchi, Angelo Paolo Dei Tos, Paolo Giovanni Casali and Roberta Sanfilippo

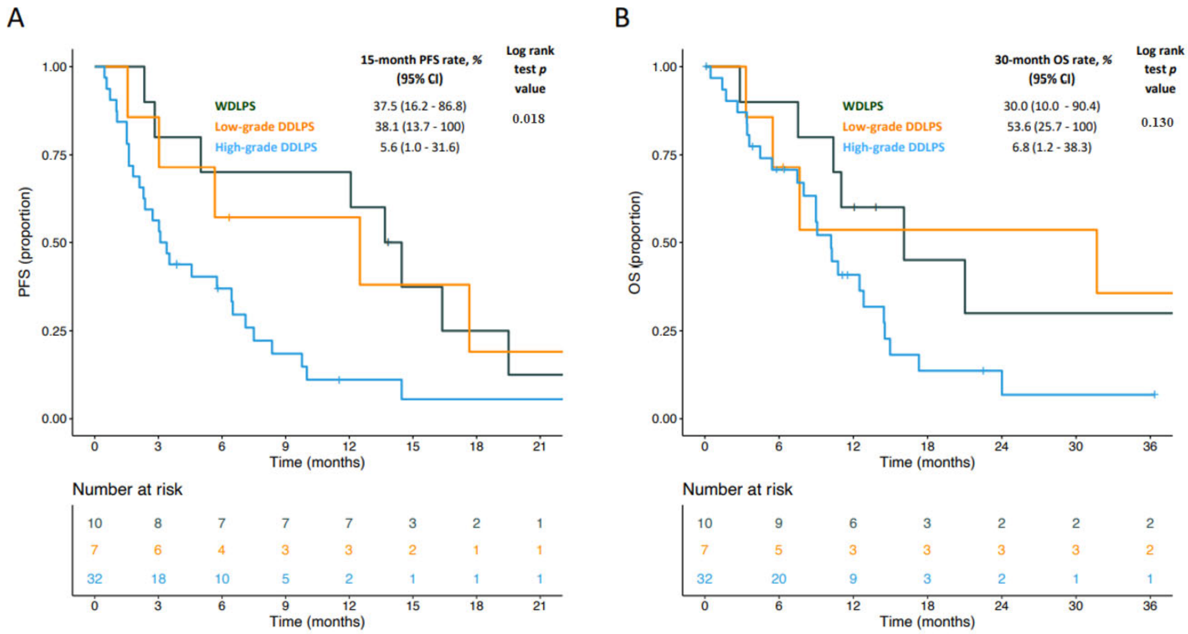

Figure S1. Kaplan–Meier curves for progression-free survival (PFS) (**A**) and OS (**B**) for well differentiated liposarcoma (WDLPS), low-grade dedifferentiated liposarcoma (DDLPS), and high-grade DDLPS. Dark green lines indicate patients with WDLPS, orange lines indicate patients with low-grade DDLPS, and light blue lines indicate patients with high-grade DDLPS.

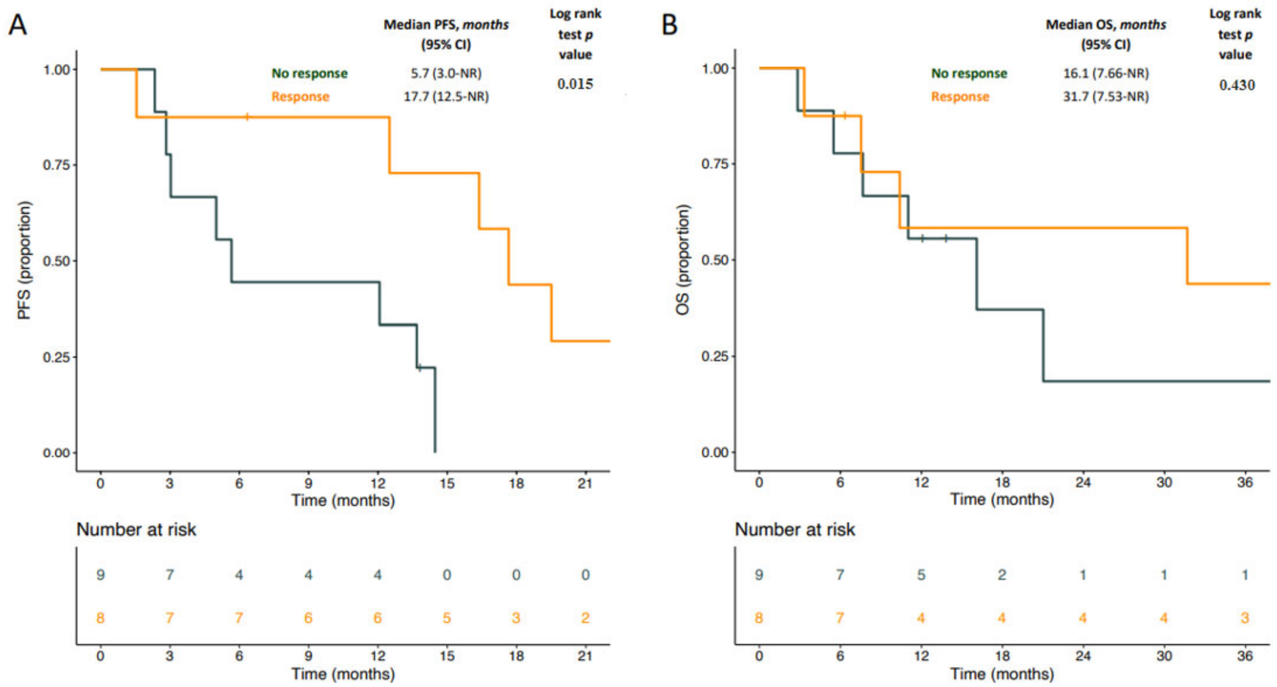

Figure S2. Kaplan–Meier curves for PFS (**A**) and overall survival (OS) (**B**) in the low-grade cohort according to overall response to trabectedin. Dark green lines indicate patients who did not achieve a tumor response, whereas orange lines indicate patients who achieved a tumor response.

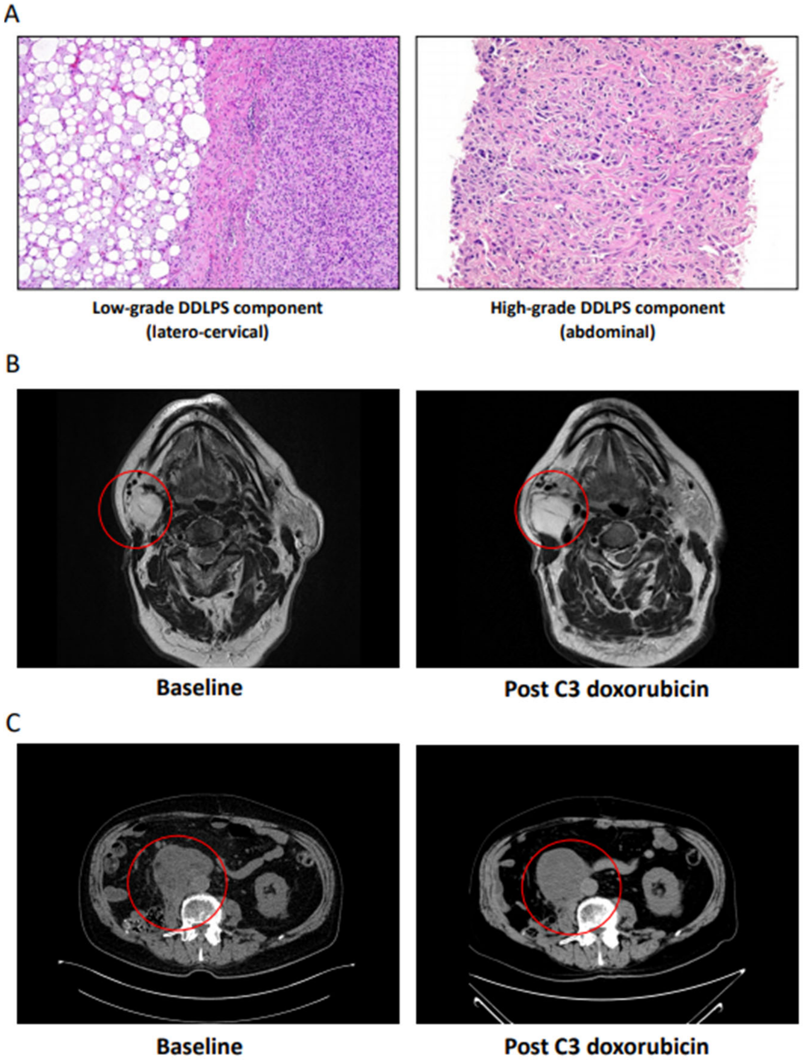

Figure S3. Histological characterization and response to doxorubicin in a patient with double DDLPS component. (**A**) shows the hematoxylin and eosin stain of tumor samples from the low-grade DDLPS laterocervical localization and from the high-grade DDLPS abdominal localization. (**B**) (T2 weighted magnetic resonance imaging) and (**C**) (computerized tomography scan) show the RECIST tumor response of the low-grade, laterocervical component and of the high-grade, abdominal component, respectively.

Table S1. Cox proportional hazard regression models for OS.

**Caracteristics.**	**Univariate analysis**		**Multivariable Model**	
	**HR (95% CI)**	***p*-Value**	**HR (95% CI)**	***p*-Value**
**Age, year**<br>≤65<br>>65	Ref<br>0.38 (0.18–0.79)	0.010	-	-
**Sex**<br>Male<br>Female	Ref<br>0.78 (0.39–1.55)	0.480	-	-
**ECOG PS**<br>0<br>1–2	Ref<br>1.13 (0.56–2.29)	0.730	-	-
**Disease extension**<br>Locally advanced<br>Metastatic	Ref<br>0.96 (0.48–1.90)	0.889	-	-
**Primary site**<br>Retroperitoneal<br>Other	Ref<br>2.71 (0.80–9.17)	0.110	-	-
**Previous lines of treatment, *n***<br>≤2<br>>2	Ref<br>1.29 (0.63–2.63)	0.493	-	-
**Grading**<br>High grade cohort<br>Low-grade cohort	Ref<br>0.46 (0.22–0.99)	0.048	Ref<br>0.55 (0.25–1.19)	0.126

Abbreviations. HR: hazard ratio; CI: confidence interval; Ref: reference; ECOG PS: Eastern Cooperative Oncology Group Performance Status.
